# Dynamic Changes in Microbial Communities and Physicochemical Characteristics During Fermentation of Non-post Fermented Shuidouchi

**DOI:** 10.3389/fnut.2022.926637

**Published:** 2022-06-13

**Authors:** Yuyong Chen, Feng Qin, Mingsheng Dong

**Affiliations:** ^1^College of Food Science and Technology, Nanjing Agricultural University, Nanjing, China; ^2^Jiangsu Agri-Animal Husbandry Vocational College, Taizhou, China

**Keywords:** non-post fermented Shuidouchi, microbial diversity, biogenic amines, alkylpyrazines, metabolism pathway, functional compounds

## Abstract

Non-post fermented Shuidouchi is a Chinese spontaneously fermented soybean food with multifunctionality in human health. The functionality and safety of this plant-based food will be affected by the microorganisms during fermentation. In this study, microbial diversity was investigated using culture-dependent and culture-independent methods. The functional metabolites such as polyamines and alkylpyrazines were also determined at different time points during fermentation. We found that *Bacillus* was the most dominant microbe throughout the fermentation process, while the temperature was the most important influencing factor. During fermentation, the microbial diversity increased at a moderate temperature and decreased at a high temperature (52^°^C). High temperature caused the prosperity of the spore-producing bacteria such as *Bacillus* (more than 90% relative abundance in bacteria) and *Aneurinibacillus* (2% or so relative abundance in bacteria), and the inhibition of fungi. Furthermore, it was found by correlation analysis that the relative abundances of *Bacillus* and *Aneurinibacillus* were positively correlated with the relative content of amino acid metabolism pathway and the content of most alkylpyrazines and biogenic amines. Meanwhile, the relative abundances of many non-dominant bacteria were negatively correlated with the content of biogenic amines and positively correlated with the relative content of carbohydrate metabolism pathway. These effects were helpful to control the biogenic amine contents under the safety limits, increasing the alkylpyrazine type and product functionality. A two-stage temperature control strategy—a moderate temperature (35–42^°^C) first, then a high temperature (52^°^C)—was concluded from the spontaneous fermentation of non-post fermented Shuidouchi. This strategy could improve the safety of product by inhibiting or sterilizing the thermolabile microbes. The non-post fermented Shuidouchi product is rich in functional compounds such as polyamines and alkylpyrazines.

## Introduction

Shuidouchi is a traditional fermented soybean product widely distributed in China (1). Previous research found that Shuidouchi is highly digestible and absorbable (2), and is rich in nutritional and functional compounds such as proteins, peptides, and active isoflavones (3, 4). Though the process may vary among different regions, there are two main types of traditional spontaneously fermented Shuidouchi. The first type involves fermenting boiled or steamed soybeans until it becomes the finished product, commonly known as mucous sauce beans or stinky Douchi, using insulation, starting with a warm temperature. The second type involves further fermenting the previous product for a ripening period after adding the boiled soybean soup and condiments such as spices and salt, without the use of insulation (3). The first type of product in this article was denoted as non-post fermented Shuidouchi, similar to Natto, with a mucous appearance and a shorter production cycle compared to the second type of product. Shuidouchi offers many health benefits, such as gastric injury prevention (4), as well as anti-oxidation (5), *in vitro* anticancer, and antimutagenic effects (6). Because non-post fermented Shuidouchi has shown vascular protective and anti-hypertension functionality (7, 8), as well as a high activity of fibrinolytic enzymes (9), with lower salt content, a shorter processing cycle, and a lower cost, it has attracted increasing attention from researchers and enterprises.

Although the fermentation process of non-post fermentation Shuidouchi is relatively short (48–72 h), various microorganisms secrete a variety of enzymes in the product that will promote the hydrolysis of soybeans and the generation of various metabolites, resulting in the appearance of a brown mucous, a Douchi smell, and a spontaneous increase in temperature. Previous research has focused on comparative studies of bacterial diversity in the initial and final stages of Shuidouchi (10) and the microbial diversity of products from different regions (11). However, there is still a lack of detailed research on the relationship of microbes and functional metabolites during the fermentation of non-post fermented Shuidouchi. Recently, the high-throughput nucleotide sequencing technique was applied to analyze the microbial diversity in many foods, as it can identify unculturable microbes (11–14). However, the culture-dependent method should also be used to investigate the microbial ecology for more accurate results. A variety of physiologically active substances such as biogenic amines (BAs) were found to generate during the fermentation of non-post fermented Shuidouchi, among which polyamines were found to exhibit protective effects against chronic diseases (15, 16). In recent years, studies have found that the content of certain BAs reached or approached harmful levels in commercial Natto and Shuidouchi products. Certain *Bacillus subtilis* strains in Natto were found to accumulate high concentrations of β-phenylethylamine and tyramine, which could cause food safety problems; thus, BAs must be monitored during fermentation (16, 17). In addition, other physiologically active substances such as alkylpyrazines were also found in fermented soybean products. Among the alkylpyrazines, 2,3,5,6-tetramethylpyrazine (tetramethylpyrazine) has been shown to promote human cardiovascular and cerebrovascular health (18–20), increase immune organ indices and natural immunoglobulins (21), relieve symptoms of animal experimental autoimmune myasthenia gravis (22), and protect against acute alcoholic liver injury (23). Tetramethylpyrazine has also been found in mold type Douchi (24, 25). These metabolites were shown to be related to the population changes and physiological metabolic pathways of microbes during fermentation. However, the correlation between microbes and alkylpyrazines in Douchi (including non-post fermented Shuidouchi) remains unknown. Thus, an investigation into the relationship of functional compounds and microbial metabolism pathways in Douchi is needed.

This research explored the microbial community structure of non-post fermented Shuidouchi, and its associations with both the functional metabolites and microbial metabolism pathways, to provide information for optimizing the safe and functional qualities of this product.

## Materials and Methods

### Chemicals and Materials

The study material consisted of yellow soybeans [*Glycine max* (Linn.) Merr.], which were purchased from a market in Yantai city, Shandong province, China.

BAs or amine hydrochlorides, such as histamine (HIS), β-phenylethylamine (PHE), Tryptamine (TRP), cadaverine dihydrochloride (CAD), putrescine dihydrochloride (PUT), tyramine hydrochloride (TYR), spermine (SPM), and spermidine (SPD), were purchased from Shanghai Yuanye Bio-Technology Co., Ltd. (Shanghai, China), with purities of ≥ 98%.

Acetone and acetonitrile were of HPLC grade, while the other chemicals were analytically pure. All chemicals were purchased from Sinopharm Group Co., Ltd. (Beijing, China), and the plate count agar (PCA, CM101), De Man Rogosa Sharpe agar (MRSA, CM188), Manitol salt agar (MSA, CP813), brain heart infusion agar (BHIA, CM918), potato dextrose agar (PDA, CM123, and yeast extract glucose chloramphenicol agar (YGC, CM840) were purchased from Land Bridge Technology Co., Ltd. (Beijing, China). Bacterial and yeast genome extraction kits and lyticase were purchased from Tiangen Biotech Co., Ltd. (Beijing, China), and the 2 × Taq master mix (P111-03) was purchased from Vazyme Biotech Co., Ltd. (Nanjing, China).

### Non-post Fermented Shuidouchi Fermentation and Sampling

**FIGURE 1:**
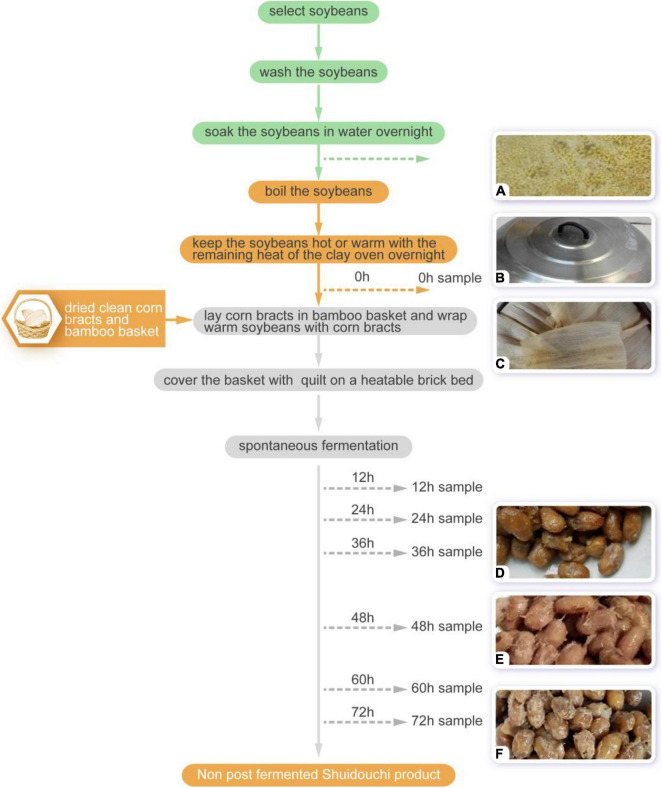
The non-post fermented Shuidouchi process and sampling time points. **(A)** Boiled soybeans; **(B)** the soybeans were kept warm with the lid covered; **(C)** the soybeans were wrapped in corn husks; **(D)** mucus appeared at 36 h; **(E)** ropy appearance of the product 48 h; **(F)** 72-h product.

The spontaneous fermentation steps for non-post fermented Shuidouchi were similar to the methods described by Chen et al. and were as follows (Figure 1): (1) the soybeans were selected; (2) the impurities were removed and the soybeans were washed twice; (3) the soybeans were soaked in water overnight; (4) then the soybeans were cleaned and boiled; (5) corn bracts were washed together with the bamboo basket after trimming and moderately dried on a hot pot cover; (6) the soybeans were kept hot or warm with the remaining heat from the clay oven overnight to turn them from yellow to yellow-red; (7) the corn bracts were spread on the bottom of a bamboo basket and the warm (35–42^°^C) cooked soybeans were scooped into the basket, where the soybeans were wrapped with corn bracts and the basket was covered with cotton clothes and a quilt on a Kang (a heatable brick bed) to adjust the soybean temperature to slightly lower than body temperature, and to start the spontaneous fermentation process; and (8) fermentation was maintained (generally for 2–3 days) until the soybeans could be removed from the mucus filaments, which were soft with a Douchi aroma (9).

Samples were collected after 0, 12, 24, 36, 48, 60, and 72 h of fermentation. About 400 g soybeans were taken out from different positions of soybean pile, mixed thoroughly and split into two parts by sterile operation. All samples were packed in sterilized containers, transported to the laboratory at –10^°^C, and stored at –70^°^C.

### Sensory Evaluation and Determination of the Temperature and pH Value

Before sampling, the core temperature of the Shuidouchi was detected by a sterilized thermometer and recorded. Then, sensory evaluation was carried out after sampling, which was described according to the Chinese standard of Natto (26). The sensory features such as color, flavor, and texture were recorded in time. The pH values of the soybean surface materials in the sample were determined using a pH meter (Mettler EL20, Switzerland) according to the standard method (27, 28).

### Enumeration of Bacteria and Fungi

Enumeration was performed according to the colony counting method using the gradient dilution spread-plate technique, similar to the procedure described by Chen et al. (9). PCA and MRSA plates were used to enumerate the bacteria at 37^°^C under micro aerobic or anaerobic conditions. The MSA plates were incubated micro-aerobically at 37^°^C to count the NaCl tolerant bacteria. In this process, BHIA, basal medium agar (BMA) (consisting of 5.0 g of yeast extract, 5.0 g of tryptone, 1.0 g of KH_2_PO_4_, 0.4 g of MgCl_2_⋅6H_2_0, 1.0 g of NH_4_Cl, 5.0 mg of FeSO_4_⋅7H_2_O, 1.0 g of glucose, 25 mL of a mineral solution, 1 mL of Tween 80, 16 g of agar for 1 L of the medium, where the pH was adjusted to 7.0 with 10 M KOH) (29), and CESP agar (CESPA) (15 g of casitone, 5 g of yeast extract, 3 g of soytone, 2 g of peptone, 0.015 g of MgSO_4_, 0.007 g of FeCl_3_, 0.002 g of MnCl_2_, 20 g of agar for 1 L medium, at a pH of 7.2) (30) plates were cultured aerobically at 50^°^C to count the thermophilic bacteria. The PDA plates and YGC plates, which were used to count the fungi, were cultured aerobically at 28^°^C. Micro-aerobic conditions were achieved by sealing the Petri dishes in a plastic bag. The plates were generally incubated for 2–3 days, while the plates without the appearance of colonies were incubated and observed for 5 days.

### DNA Extraction, PCR Amplification, and High-Throughput Nucleotide Sequencing

For bacterial total genome DNA extraction, we obtained 20 g of the Shuidouchi sample and added 180 mL of the sterile physiological saline (40 g of 0 h sample and 160 mL of sterile physiological saline) in a sterile conical flask with a cover, which was shaken at 4^°^C for 30 min. The solution was then centrifuged for 15 min at 4°C and 2,000 g, and the supernatant was centrifuged for 20 min at 4^°^C and 12,000 g. Then, the supernatant was discarded, and the precipitate was placed into a sterile 1.5 mL EP tube to extract the DNA with a bacterial genome extraction kit (31). During fungal genome extraction, the same method was used; however, during the second centrifugation, the supernatant of the first centrifugation was centrifuged for 20 min at 14,000 g. The fungal precipitate was treated with lyticase, and then extracted with the yeast genome extraction kit (32).

Regions V3 to V4 of the 16S rDNA were amplified using primers (341F, 5′-(CCCTACACGACGCTCTTCCGATCTG) (barcode) CCTACGGGNGGCWGCAG-3′, 805R, 5′-(GACTGGAGTTCCTTGGCACCCGAGAATTCCA) (barcode) GACTACHVGGGTATCTAATCC-3′) with the barcode. The fungal internal transcribed spacer (ITS) regions ITS1-ITS2 were amplified using primers (ITS1F, 5′- (CCCTACACGACGCTCTTCCGATCTN) (barcode) CTTGGTCATTTAGAGGAAGTAA-3′, ITS2R, 5′-(GTGACTGGAGTTCCTTGGCACCCGAGAATTCCA) (barcode) GCTGCGTTCTTCATCGATGC-3′). Amplification was performed in a reaction volume of 30 μL containing 15 μL of 2 × Taq master mix, with 1 μL of each primer (10 μM), 10–20 ng of the DNA and dd-H_2_O samples, according to the following procedures. Initialization was conducted at 94^°^C for 3 min, with 5 cycles of 30 s at 94^°^C, 20 s at 45^°^C, and 30 s at 65^°^C. Then, 20 cycles consisting of 20 s at 94^°^C, 20 s at 55^°^C, and 30 s at 72^°^C, with an extension step for 5 min at 72^°^C, were undertaken. The PCR products were stored at 10^°^C before the second round of PCR.

Illumina bridge PCR compatible primers were introduced into the second round of PCR, where the reaction was performed in a 30 μL mixture containing 15 μL of 2 × Taq master mix, 1 μL of primer F (10 μM), 1 μL of primer R (10 μM), 20 ng of the last round of the PCR and dd-H_2_O products, using the following procedures. Initialization was conducted at 95^°^C for 3 min, with 5 cycles of 20 s at 94^°^C, 20 s at 55^°^C, and 30 s at 72^°^C. Then, an extension step at 72^°^C was conducted for 5 min, and cooled down to 10^°^C. The PCR products were purified by MagicPure size selection DNA beads (TransGen Biotech, Beijing, China), and pooled in equimolar and then paired-end sequenced on an Illumina MiSeq PE300 platform (Illumina, San Diego, United States), according to the standard protocols by Sangon Biotech Co., Ltd. (Shanghai, China).

### Bioinformatics Data Processing and Analysis

Operational taxonomic units (OTUs) were clustered with a 97% similarity cutoff using Usearch (version5.2.236),^1^ while the chimeric sequences were identified and removed using UCHIME (version 4.2.40).^2^ The taxonomy of each 16S rRNA and ITS rRNA gene sequence was analyzed by an RDP Classifier algorithm^3^ against the RDP,^4^ Silva bacterial^5^ and Unite fungal databases,^6^ using a confidence threshold of 0.8. α- and β-diversity, as analyzed by R and Mothur software.^7^ Principle coordinate analysis (PCoA) was carried out on the Unifrac metric with the vegan (version 2.0-10) package of the R language. The relationships between the bacterial or fungal communities of samples and environmental factors (pH, temperature, bioamine, 2,3-butanediol and tetramethylpyrazine) were explored by canonical correspondence analysis (CCA) using the vegan (version 2.0-10) package of the R language. The correlation between microbes and physical or chemical characteristics was evaluated by Pearson correlation analysis. The Kyoto Encyclopedia of Genes and Genomes (KEGG) pathway information was obtained by the Phylogenetic Investigation of Communities by Reconstruction of Unobserved States (PICRUSt) software (33).

### Determination of Biogenic Amines in the Samples

BA determination was performed according to the methods described in the national standards in China (34).

According to Han’s method, 5 g of each ground sample was placed into 50 mL capped centrifuge tubes, with 20 mL perchloric acid solution (0.4 mol/L) added, before mixing and shaking for 30 min. Each mixture was centrifuged at 7,000 rpm for 6 min at 4^°^C, and the supernatant was collected. Then the precipitate was extracted according to the above methods. The supernatant was extracted twice and then combined to fill 50 mL with perchloric acid solution. Then, 1 mL of the mixed solution was obtained for derivatization (17).

Next, 1 mL of each extracted sample or standard BA solution was homogeneously mixed with 200 μL of 2 M NaOH solution, 300 μL of saturated NaHCO_3_ solution and then 1 mL of dansyl chloride solution (10 mg/mL in acetone) in a 5 mL centrifuge tube. The homogenized mixture was incubated in a water bath at 60^°^C for 30 min, protected from light, and sufficiently shaken every 15 min. Then, 100 μL of 25% ammonia solution was added into the mixture and blended to stop the reaction. After 30 min, the reaction solution was blended with 700 μL of acetonitrile. The final solution was filtered through a 0.22-μm filter, and the filtrate was maintained at 4^°^C for HPLC inspection (17).

Quantitative analysis of the BAs was carried out using an HPLC (1260 Infinity, Agilent, United States) with a UV detector and acid resistant liquid chromatography column (ZORBAX SB-Aq 4.6 × 250 mm, 5 μm, Agilent, United States). The mobile phase solvent A was dd-H_2_O, while solvent B was acetonitrile. The gradient elution program followed the ratio of A:B, with 100:0 at 0 min, which decreased gradually to 30:100 at 5 min and 0:100 at 16 min, and then increased to 35:65 at 18 min and maintained at 35:65 until 30 min. The flow velocity of the previous 5 min was 5 mL/min, and then adjusted to 0.8 mL/min. The temperature of the column was 30^°^C, with 20 μL of injected sample and a UV detection wavelength of 254 nm.

### 2,3-Butanediol, Acetoin and Alkylpyrazine Determination

The solid phase microextraction method (SPME) was used to analyze 2,3-butanediol, acetoin, and alkylpyrazine according to the methods described by Zhang et al. (24), with minimal modifications. First, 3 g of the sample was put into a 15 mL headspace vial, equilibrated for 10 min at 60^°^C. Then the volatile components were extracted using a 50/30 μm divinylbenzene/carboxen/polydimethylsiloxane (DVB/CAR/PDMS, Supelco, Bellefonte, PA, United States) fiber for 30 min at 60^°^C, which was conditioned in a GC injector port at 250^°^C for 2 h prior to use. After extraction, the volatiles that adsorbed on the SPME fibers were thermally desorbed at 230^°^C in the injection port of an Agilent 7890B gas chromatography system (Agilent Technologies, United States) coupled to an Agilent 5977A quadrupole inert mass selective detector by holding it in splitless mode for 3 min. An Hp-5 fused silica capillary column (30 m × 0.32 mm I.D. × 0.25 μm film thickness) with helium flow at 1.2 mL/min was used to separate the volatile components. The GC condition consisted: the injector at 230^°^C, initial column temperature of 20^°^C for 5 min, which was increased to 30^°^C at a rate of 2^°^C/min, maintained at 30^°^C for 3 min, further increased to 150^°^C at a rate of 3^°^C/min, then increased to 250^°^C at a rate of 10^°^C/min and maintained at 250^°^C for 10 min. The MS condition was: 250^°^C of transfer line, 230^°^C of electron ionization (70 eV), where the temperature of the quadrupole mass analyzer was 150^°^C, and at full scanning mode (35–550 m/z). The compounds were determined by comparing with the NIST 14.L database. The relative content of 2,3-butanediol, acetoin and alkylpyrazine was quantified by area normalization.

### Statistical Analysis

The relationships between the relative abundance of microbial communities and the chemical/physical characteristics were analyzed using R language (V 4.1.2), by calculating the Pearson correlation coefficient. Duncan multiple comparison of the data was provided by mean comparisons using one-way variance analysis using IBM SPSS Statistics 26 with a level of significance of *p* < 0.05 when the variances were homogeneous. When the variances were not homogeneous, Games-Howell comparison was used.

## Results

### Changes in Temperature, pH Value, Sensory Characteristics, and Microbial Growth

During fermentation of the non-post fermented Shuidouchi, the temperature increased spontaneously (Table 1). In the early 12 h, the temperature increased slowly and the soybeans were maintained in a warm state, while the temperature increased sharply to 52^°^C between 12 and 24 h during the initial fermentation stage and remained as high as approximately 51^°^C during fermentation metaphase. In the next fermentation phase (anaphase), the temperature decreased slowly but was still maintained at a high level, and the pH of the soybeans increased significantly after 12 h. The increase in temperature also brought about changes in the sensory characteristics. During the moderate temperature fermentation stage (0–24 h), a sour and mellow aroma arose from the fermented soybeans at 12 h, accompanied by the growth of mesophilic bacteria, yeast, and mold (8.10, 4.09, and 2.78 log CFU/g) (Table 2, 12 h). Then, the high temperatures throughout the metaphase and anaphase of fermentation also brought about a dark color of the soybeans (Table 1 and Figure 1), mucus filaments, and a Douchi flavor (Table 1). After 72 h of fermentation, the pH value reached 8.10, and the taste of the product would be more bitter if the fermentation was not terminated. Thus, the optimal fermentation time was 48–72 h.

**TABLE 1:** Changes in the pH value, temperature, and sensory characteristics of the non-post fermented Shuidouchi during fermentation.

Characteristic	Fermentation time/hours						
	Initial stage			Metaphase		Anaphase	
	0	12	24	36	48	60	72
Temperature/^°^C	41.93 ± 0.06^d^	42.33 ± 0.58^d^	52.17 ± 0.76^a^	51.87 ± 0.23^a^	50.67 ± 0.58^ab^	47.50 ± 0.87^bc^	44.67 ± 0.58^cd^
pH	6.30 ± 0.02^f^	6.36 ± 0.01^f^	6.48 ± 0.01*^e^*	7.43 ± 0.01^d^	7.79 ± 0.00^c^	7.85 ± 0.01^b^	8.10 ± 0.01^a^
Color	Yellow with red	Yellow with red	Yellow with red	Brownish orange	Brownish orange	Brownish orange	Light brown
Flavor	Cooked soybean flavor	Sour and mellow	Slight Douchi flavor	Douchi flavor	Strong Douchi flavor	Strong Douchi flavor	Strong Douchi flavor
Texture	Soft, without mucus	Soft, without mucus	Soft, short mucus filaments	Soft, long mucus filaments	Softer, a lot of mucus and long mucus filaments	Softer, a lot of mucus and long mucus filaments	Softer, the mucus thickened and filaments decreased

*Trials were conducted in triplicate. ^a–f^Values in the columns with different lowercase letters were considered significantly different (p < 0.05).*

**TABLE 2:** Evolution of microbial population in the non-post fermented Shuidouchi during fermentation (unit was log CFU/g).

Culture medium	Culture conditions	Fermentation time/hours						
		Initial stage			Metaphase		Anaphase	
		0	12	24	36	48	60	72
**Bacteria**								
PCA	37^°^C, micro aerobic	2.91	8.10	8.10	8.13	8.20	8.40	8.58
	37^°^C, anaerobic	2.30	8.09	7.84	7.97	8.11	8.02	8.48
MRSA	37^°^C, micro aerobic	2.00	7.47	8.00	6.72	6.81	7.08	6.78
	37^°^C, anaerobic	2.00	7.62	8.09	6.46	7.43	7.21	8.28
MSA	37^°^C, micro aerobic	<2.00	6.30	7.28	6.60	7.45	7.45	8.30
BHIA	50^°^C, aerobic	<2.00	6.66	8.26	8.78	9.58	10.30	10.66
BMA	50^°^C, aerobic	<2.00	4.90	8.30	9.28	9.53	10.28	9.90
CESPA	50^°^C, aerobic	<2.00	5.72	8.48	9.11	9.00	9.00	9.38
**Yeast**								
PDA	28^°^C, aerobic	<1.70	4.03	<3.00	<2.00	<2.00	<2.00	2.86
YGC	28^°^C, aerobic	<1.70	4.09	<3.00	-	-	-	-
**Mold**								
PDA	28^°^C, aerobic	<1.70	2.78	<3.00	<2.00	<2.00	<2.00	<2.00
YGC	28^°^C, aerobic	<1.70	2.66	<3.00	-	-	-	

*“-”, undo.*

According to the colony counting results, we found that there were low counts of mesophilic bacteria at the starting point (Table 2, 0 h) of fermentation; however, there were fewer thermoduric bacteria, yeast, and molds with counts below the detection limit. In the first 12 h of fermentation, due to the appropriate temperature and pH, the mesophilic bacterial counts were as high as 10–100 times those of the thermoduric bacterial counts. After 12 h, the thermoduric bacteria gradually increased. At 24 h, the thermoduric bacterial counts increased to the same level as the mesophilic bacteria, and then gradually surpassed the mesophilic bacteria. At the end of fermentation, the thermoduric bacterial counts reached 10–100 times those of the mesophilic bacteria. The fungi were detected only 12 h in the initial stage of fermentation, indicating that the yeast and mold grew at a moderate temperature, though the yeast counts were slightly higher. The bacteria were the most dominant, and the yeast also occupied a certain position; however, the mold was inhibited. The high temperatures during metaphase and anaphase of fermentation reduced the counts of yeast and mold below the detection limit (< 2 log CFU/g). However, the temperature drop (45^°^C) at the end of fermentation allowed the yeast to recover and proliferate, and the mold was not detected under the combined effect of high temperature and pH. We concluded that the bacteria occupied the most dominant position during the fermentation of non-post fermented Shuidouchi, and the fungi only proliferated during the early and end stages without a dominant position throughout the fermentation process, while the products were dominated by the thermoduric bacteria.

### Microbial Diversity of the Non-post Fermented Shuidouchi

#### Alpha Diversity of the Fermentation Samples

In this study, 335,947 bacterial and 295,019 fungal high-quality reads were obtained from the non-post fermented Shuidouchi samples (Table 3), where the average of the bacterial and fungal reads in the samples were 47,992 and 42,145, respectively. In addition, the Good’s coverages of all samples were more than 0.998 (Table 3) with a similarity level of 97%, proving an effective cover for the microbial species. Furthermore, 592 OTUs were obtained from the bacterial reads, which were clustered into 15 phyla and 119 genera, and 2,809 OTUs were obtained from fungal reads belonging to 15 phyla and 377 genera. Indices of alpha diversity such as Chao1, Shannon, and Good’s coverage (Coverage) are also listed in Table 3. The bacterial Shannon index increased first and then gradually decreased, and was the highest at a time point of 12 h. During fermentation, the bacterial Shannon index gradually decreased with increasing temperature and pH (Tables 1, 3), and the fungal Shannon index was greater than the bacteria. During fermentation, the fungal Shannon index increased slightly after 12 h of fermentation and remained stable; however, with increasing temperature and pH, it decreased sharply after 60 h of fermentation. It increased again after 72 h at the end of fermentation with decreasing temperature (Tables 1, 3).

**TABLE 3:** Sequence number and alpha diversity indices of bacterial and fungal community of the non-post fermented Shuidouchi samples.

Sample	16S rDNA gene				ITS rDNA gene			
	Sequence number	Shannon	Chao1	Coverage	Sequence number	Shannon	Chao1	Coverage
0 h	61,606	0.20	131.85	0.999	40,978	4.98	966.65	0.998
12 h	42,239	1.70	222.03	0.999	39,782	5.13	906.15	0.998
24 h	53,141	2.14	264.50	0.999	39,895	5.14	974.67	0.998
36 h	46,327	1.73	341.06	0.998	50,440	5.14	1050.55	0.998
48 h	26,926	1.24	202.96	0.998	32,319	5.01	891.06	0.998
60 h	50,228	1.14	222.75	0.999	55,840	1.60	772.94	0.998
72 h	55,480	1.13	282.77	0.999	35,765	5.16	892.13	0.998

#### Venn Diagrams of Bacteria and Fungi in Samples

**FIGURE 2:**
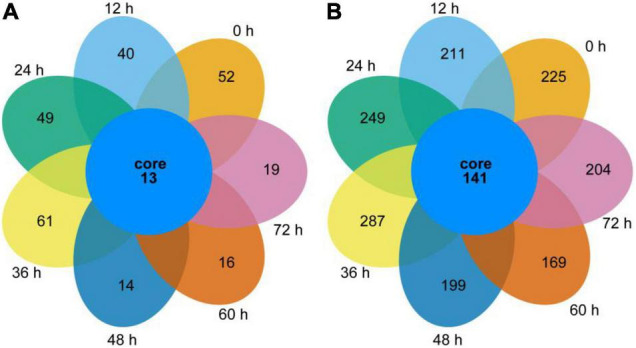
Bacterial and fungal Venn diagrams of the non-post fermented Shuidouchi. **(A)** Venn diagrams of the bacterial OTUs, **(B)** Venn diagrams of the fungal OTUs.

As shown by the Venn diagram in Figure 2A, a total of 592 bacterial OTUs were detected in all samples, and 13 shared OTUs were found. The number of unique OTUs in the 0, 12, 24, and 36 h samples was larger than in the 48, 60, and 72 h samples. The common OTUs were allocated to *Bacillus* (30.77%), *Acinetobacter* (23.08%), *Pantoea* (15.38%), *Enterococcus* (7.69%), *Lactococcus* (7.69%), *Citrobacter* (7.69), and *Klebsiella* (7.69%) in the genus level.

As shown in Figure 2B, 2,809 fungal OTUs were observed in the diagram, which was significantly more than the bacterial counterpart. Thus, 141 common OTUs were allocated to unclassified (20.57%), unclassified Fungi (6.38%), *Aspergillus* (4.26%), *Mortierella* (3.55%), *Tomentella* (2.84%), unclassified *Sordariomycetes* (2.84%), *Lactarius*, *Sarocladium*, unclassified *Ascomycota*, unclassified *Lasiosphaeriaceae* (each of the former 4 genera 2.13%), *Alternaria*, *Candida*, *Fusarium*, unclassified *Hypocreales*, unclassified *Pleosporales*, unclassified *Rozellomycota*, unclassified *Sordariales*, *Verticillium* (each of the former 8 genera 1.42%), and other 56 genera (each 0.71%) in the genus level. The number of unique OTUs in each sample was 225, 211, 249, 287, 199, 169, and 204 for 0, 12, 24, 36, 48, 60, and 72 h, respectively.

These results suggested that a higher fermentation temperature coupled with a higher pH reduced the number of unique microbes and microbial diversity of the samples in the second half of fermentation.

#### Changes in the Microbial Communities of the Non-post Fermented Shuidouchi

**FIGURE 3:**
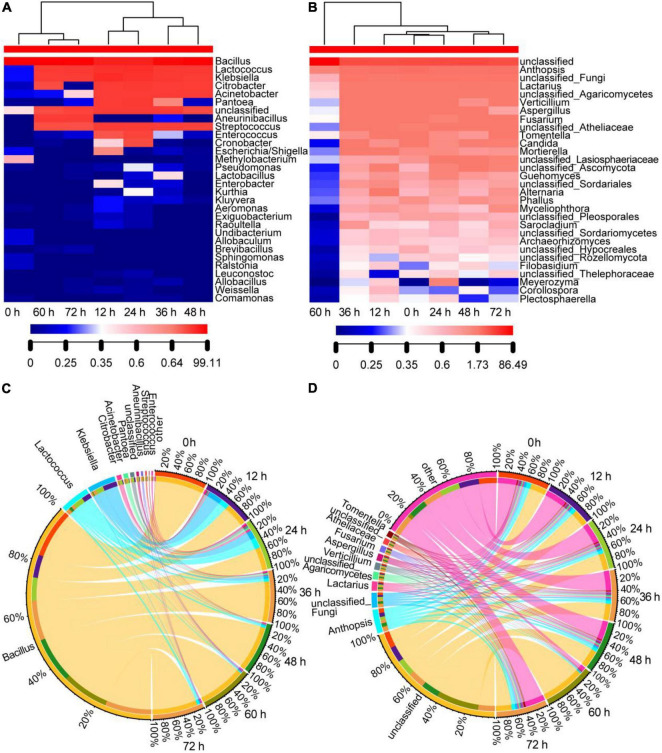
Genera distribution of the bacterial and fungal communities in the non-post fermented Shuidouchi: **(A)** Bacterial community heatmap (top 30 genera); **(B)** fungal community heatmap (top 30 genera), where the number indicates the value of the genus relative abundance; **(C)** the Circos of the bacterial genera distribution in the samples; **(D)** the Circos of the fungal genera distribution in the samples, where the relative abundance of the genera is represented by the relative tick on the outer circle, and the relative abundance of the genera is represented by the thickness of the colorful ribbons.

The changes in bacterial community composition are shown in Figures 3A,C. We observed the lowest level of richness and evenness at the starting point of fermentation (0 h), and the evenness increased at 12 and 24 h in the initial stage and decreased in the metaphase and anaphase stages (36, 48, 60, and 72 h). At a time point of 12 h, the number of genus types, and the relative abundance of genera in the orders of *Enterobacterales* and *Pseudomonadales* were high, mainly including *Klebsiella*, *Citrobacter*, *Acinetobacter*, *Pantoea*, *Cronobacter*, *Escherichia*/*Shigella*, and *Pseudomonas*. In the metaphase stage of fermentation, only *Klebsiella*, *Citrobacter*, and *Acinetobacter* remained with their relative abundance, which continued to decrease, and only *Klebsiella* remained with a low relative abundance (about 1%) at the end of fermentation (72 h). The reason for this phenomenon was that the moderate temperature during the initial stage was suitable for the growth of most types of bacteria, while the temperature after 12 h continued to remain at approximately 50^°^C and the pH gradually increased to 8.1 (Table 1), reaching the growth limit of most bacteria. *Lactococcus* increased during the initial stage of fermentation; however, it maintained a low relative abundance level of about 5% in the metaphase and anaphase stages. The relative abundance of *Aneurinibacillus* increased gradually to 2% or so in the metaphase and anaphase stages. Meanwhile, *Bacillus* had the highest abundance in all bacterial sequences, where the relative abundance was the highest in all samples and gradually increased to more than 90% during fermentation, and it was the most dominant bacterial genus throughout fermentation. The high temperature and pH reduced the bacterial diversity of the samples, resulting in clustering of the samples during anaphase and in the 0 h sample (Figure 3A).

The fungal heatmap (Figure 3B) showed that the diversity of fungi was much higher than the bacteria, and the evenness and richness of the fungi remained constant during fermentation compared to the bacterial community, except for the 60-h sample, which decreased due to the high temperature (50^°^C) and high pH (7.85) effect for 12 h. However, the richness and evenness of the fungal community returned when the temperature dropped to 45^°^C (72 h sample). The top 10 genera of classified fungi, arranged in the order of relative abundance from high to low, consisted of *Anthopsis*, unclassified Fungi, *Lactarius*, unclassified *Agaricomycetes*, *Verticillium*, *Aspergillus*, *Fusarium*, unclassified *Atheliaceae*, *Tomentella*, and *Candida*. However, the relative abundance of each genus in these classified fungi was lower than 10%, and 352 genera of all 372 classified genera had a relative abundance that was lower than 1%. The relative abundance of the top 10 genera in the different samples was similar (Figure 3D). These phenomena indicated that the fungi were not prosperous, as the fermentation temperature was higher than their growth limits.

#### Principal Coordinate Analysis

**FIGURE 4:**
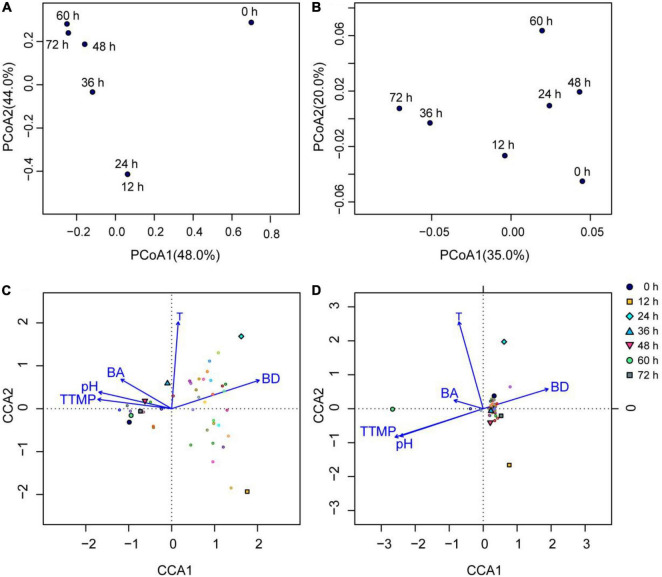
Bacterial and fungal principle coordinate analysis (PCoA) based on the weighted Unifrac distance at the OTU level, and CCA analysis based on the genus level (top 20 genera) in the non-post fermented Shuidouchi: **(A)** bacterial PCoA; **(B)** fungal PCoA; **(C)** the CCA of the bacterial communities in the samples; **(D)** CCA of the fungal communities in the samples, where T, temperature; BA, total biogenic amines concentration; BD, 2,3-butanediol.

As shown by the bacterial PCoA (Figure 4A), the samples were regularly clustered based on the evolutionary distance (weighted Unifrac distance) of the bacterial OTUs, where the 12 and 24 h samples were clustered, while the 36, 48, 60, and 72 h samples were also grouped, and the 0 h sample was far away from the samples of the other time points. The results showed that the number and abundance of the bacterial genera were nearly the same at 12 and 24 h; however, this changed greatly after 24 h during fermentation. The number and abundance of the bacterial genera in the latter half of fermentation (48, 60, and 72 h) were similar. The clustering results shown in Figure 4A were consistent with the changes in the fermentation temperature and pH (Table 1), because the 0 h sample had the lowest temperature while the samples at the other time points had higher temperatures, and the pH increased sharply during fermentation from 24 to 72 h, which changed the evolutionary distance of the fermentation samples.

Meanwhile, the number and abundance of the fungal genera in the different samples were similar, because the distances between the samples were far shorter in Figure 4B than in Figure 4A. That indicated that the fungi did not flourish because of an unsuitable temperature and pH (Figure 4B, Section “Changes in Temperature, pH Value, Sensory Characteristics, and Microbial Growth”).

### Chemical Analysis of the Non-post Fermented Shuidouchi

The concentrations of each biogenic amine were lower than 50 μg/g (Table 4). We also found that TRP increased sharply at the end of fermentation, while PHE increased slowly from 36 to 72 h. PUT was found in the cooked soybeans, which increased rapidly during the initial stage of fermentation (24 h), and then decreased slowly. CAD gradually increased in the metaphase and anaphase stages of fermentation, and HIS appeared in the metaphase stage of fermentation (36 h) and then decreased gradually. TYR appeared at a very low concentration at 24 h, and then was maintained at a low level. In addition, SPD was originally at a high level in the cooked soybeans, and increased slightly during fermentation, while SPM was found in a small amount in the cooked soybeans and increased significantly from the metaphase stage (36 h); however, the concentration was not high. Total biogenic amine content (total concentration of 8 biogenic amines) increased significantly with time.

**TABLE 4:** Changes in the biogenic amine concentration in the non-post fermented Shuidouchi.

Biogenic amine	0 h	12 h	24 h	36 h	48 h	60 h	72 h
TRP (μg/g)	0^b^	0.44 ± 0.76^b^	0^b^	0^b^	2.09 ± 3.62^b^	0^b^	22.54 ± 14.09^a^
PHE (μg/g)	0^b^	0^b^	0^b^	1.88 ± 0.02^b^	1.06 ± 0.63^b^	1.80 ± 0.02^b^	8.44 ± 7.04^a^
PUT (μg/g)	4.93 ± 0.91^d^	5.38 ± 0.03^d^	19.01 ± 0.19^a^	12.53 ± 0.30^bc^	13.28 ± 1.13^b^	12.19 ± 0.64^bc^	11.42 ± 0.54^c^
CAD (μg/g)	0.91 ± 0.10^d^	2.28 ± 0.06^cd^	3.57 ± 0.28^c^	6.40 ± 0.23^b^	7.23 ± 0.19^b^	9.65 ± 0.06^a^	11.08 ± 3.52^a^
HIS (μg/g)	0^c^	0^c^	0^c^	2.74 ± 0.30^a^	1.51 ± 1.31^b^	0^c^	0.73 ± 1.26^bc^
TYR (μg/g)	0^d^	0^d^	0.55 ± 0.01^d^	3.34 ± 0.01^b^	1.41 ± 0.02^c^	4.81 ± 0.02^a^	2.68 ± 1.08^b^
SPD (μg/g)	8.37 ± 0.01^bc^	7.03 ± 0.01^bc^	6.52 ± 0.01^c^	8.84 ± 0.01^b^	8.10 ± 0.01^bc^	7.58 ± 0.01^bc^	11.55 ± 2.99^a^
SPM (μg/g)	3.93 ± 0.03^c^	2.60 ± 0.27^c^	3.15 ± 0.02^c^	6.52 ± 0.01^b^	5.03 ± 0.01^bc^	9.23 ± 0.02^a^	9.16 ± 3.59^a^
Total biogenic amines (μg/g)	18.14 ± 0.81^c^	17.73 ± 1.07^c^	32.80 ± 0.35^bc^	42.25 ± 0.85^b^	39.72 ± 2.47^b^	45.26 ± 0.60^b^	77.60 ± 30.45^a^

*Trials were conducted in triplicate. ^a–d^Values in the columns with different lowercase letters were considered significantly different (p < 0.05).*

Pearson correlation (r) analysis of the biogenic amines is shown in Table 5, where the r value between HIS and bioamine (total biogenic amines) was close to 0. The other seven biogenic amines had a positive correlation with bioamine; thus, bioamine could be used as an important monitoring index. Similarly, the correlation between the histamine and fermentation time was low, while the r values between the other seven biogenic amines, bioamine, and time showed a significantly positive correlation.

**TABLE 5:** Pearson correlation coefficients of the biogenic amine index during fermentation of the non-post fermented Shuidouchi.

	TRP	PHE	PUT	CAD	HIS	TYR	SPD	SPM	Bioamine	Time
TRP	1.000	0.940**	–0.007	0.654**	–0.122	0.253	0.901**	0.634**	0.873**	0.548**
PHE	0.940**	1.000	0.051	0.762**	–0.099	0.463*	0.930**	0.783**	0.929**	0.605**
PUT	–0.007	0.051	1.000	0.355	0.183	0.292	–0.136	0.137	0.324	0.432
CAD	0.654**	0.762**	0.355	1.000	0.148	0.827**	0.645**	0.915**	0.911**	0.950**
HIS	–0.122	–0.099	0.183	0.148	1.000	0.234	0.079	0.042	0.083	0.232
TYR	0.253	0.463*	0.292	0.827**	0.234	1.000	0.376	0.884**	0.637**	0.761**
SPD	0.901**	0.930**	–0.136	0.645**	0.079	0.376	1.000	0.720**	0.840**	0.490*
SPM	0.634**	0.783**	0.137	0.915**	0.042	0.884**	0.720**	1.000	0.860**	0.797**
Bioamine	0.873**	0.929**	0.324	0.911**	0.083	0.637**	0.840**	0.860**	1.000	0.817**
Time	0.548**	0.605**	0.432	0.950**	0.232	0.761**	0.490*	0.797**	0.817**	1.000

Bioamine, total biogenic amines; *P < 0.05, **P < 0.01.

The alkylpyrazines and related compounds (2,3-butanediol and acetoin) were identified and are listed in Table 6. No alkylpyrazine content was found before 12 h, and minimal alkylpyrazine content appeared at 24 h during fermentation. Between 36 and 72 h, alkylpyrazine was always the main volatile compound in the product (relative content of more than 20%), where the highest was tetramethylpyrazine, followed by trimethylpyrazine, 2,5-dimethylpyrazine (2,5-DMP), 2-ethyl-3,5,6-trimethylpyrazine (3,5,6-ETMP), and 2-ethyl-3,6-dimethylpyrazine (3,6-EDMP). Tetramethylpyrazine is also named ligustrazine (2,3,5,6-tetramethylpyrazine), which could be used as a promising remedy for cardiovascular diseases (35). The high relative content of tetramethylpyrazine during fermentation (36–72 h) of the non-post fermented Shuidouchi indicated that this product offered cardiovascular and cerebrovascular health care properties after 36 h of fermentation.

**TABLE 6:** Changes in 2,3-butanediol, acetoin, and alkylpyrazine in the non-post fermented Shuidouchi.

Compounds	Relative content (%)						
	0 h	12 h	24 h	36 h	48 h	60 h	72 h
BD	0	1.67	**2.03**	2.36	0.82	0.30	0
Acetoin	0	0	0	2.55	0	0	0.01
2,5-DMP	0	0	**0.12**	**9.07**	**15.97**	**9.76**	**8.34**
3,6-EDMP	0	0	0	**0.81**	**1.15**	**0.84**	**0.67**
TTMP	0	0	**0.04**	**16.17**	**13.99**	**23.28**	**28.99**
3,5,6-ETMP	0	0	0	**0.89**	**1.36**	**2.00**	**2.02**
TMP	0	0	0	0	0	0	**9.58**

*In this work, the following definitions were used: BD, 2,3-butanediol; 2,5-DMP, 2,5-dimethylpyrazine; 3,6-EDMP, 2-ethyl-3,6-dimethylpyrazine; TTMP, tetramethylpyrazine; 3,5,6-ETMP, 2-ethyl-3,5,6-trimethylpyrazine; TMP, trimethylpyrazine. The matching degree between the compounds corresponding to the bold black number and the NIST 14.L library was greater than or equal to 80%, while the matching degree of the corresponding compounds with the red numbers was 50–80%.*

### Correlation of Microbiota and Influence Factors in Non-post Fermented Shuidouchi

As shown in Figures 4C,D, the arrows of temperature were longer than other arrows, indicating the temperature was the most significant influential factor toward the changes in bacterial and fungal communities. The angle between the temperature and BA was acute, as it indicated a positive correlation between the temperature and total biogenic amine concentration. We observed weak positive correlations between the temperature and pH, tetramethylpyrazine, or 2,3-butanediol (Figure 4C); however, 2,3-butanediol was negatively correlated with tetramethylpyrazine, pH, and BA because the intersection angles of the arrows were obtuse (Figures 4C,D).

In CCA figures, the closer the projection point of the sample (or community structure, OTU) is to the arrow, the greater the impact of the environmental factor on the sample (or community structure, OTU). For the bacterial CCA diagram (Figure 4C), we found that temperature had a significant impact on the bacterial communities in the 24- and 36-h samples, where 2,3-butanediol had a significant effect on the bacterial communities in the 12- and 24-h samples. In addition, tetramethylpyrazine, pH, and BA had great effects on the bacterial communities of the fermentation samples at 0, 60, 72, and 48 h, while the 12- and 24-h samples had the highest relative abundance of bacterial flora on the genus level.

The temperature in the CCA diagram of the fungi (Figure 4D) had a significant impact on the fungal community in the 24- and 60-h samples, while the tetramethylpyrazine and pH had the greatest effects on the fungal community in the sample fermented for 60 h. The relative abundance of fungal flora in the 12-, 24-, and 60-h samples was higher than in the other samples.

### Pearson Correlation Between the Microbes and Chemicals in the Non-post Fermented Shuidouchi

**FIGURE 5:**
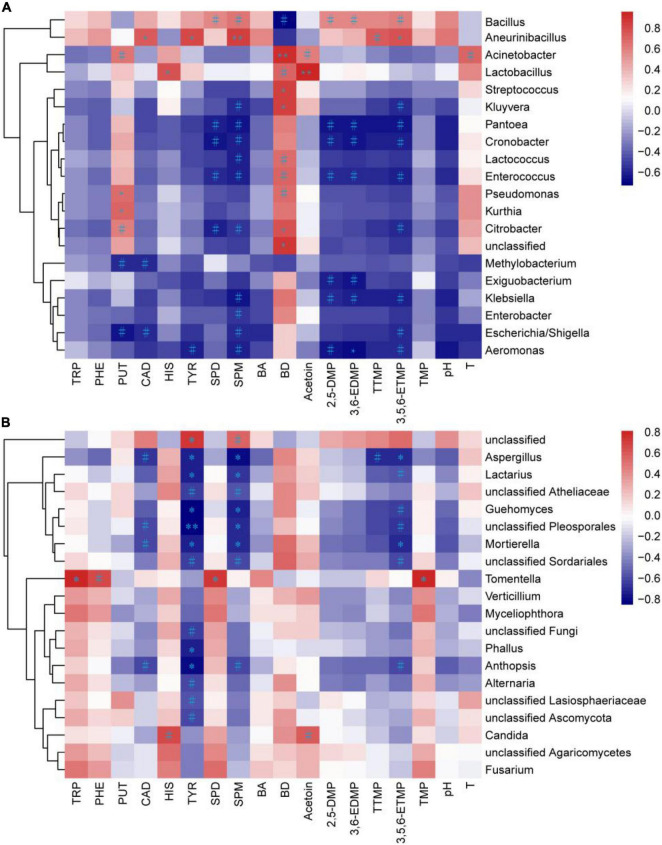
Heatmap of the correlations between the microbiota and chemical characteristics in the non-post fermented Shuidouchi samples. The Pearson correlation coefficient r in the bacterial **(A)** and fungal **(B)** communities ranged from –1 to 1; positive correlation was *r* > 0, and the negative correlation was *r* < 0, where ^#^represents *p* < 0.1, *represents *p* < 0.05 and ^**^represents *p* < 0.01, and T, temperature; BA, total biogenic amine concentration; BD, 2,3-butanediol.

The chemical characteristics, temperature, and pH were used to analyze their Pearson correlation with the bacterial and fungal abundance (top 20 genera) in the non-post fermented Shuidouchi (Figure 5). As shown in Figure 5A, there were 27 positive correlations between 20 bacterial genera and the above characteristics at a significance level of *P* < 0.1, while 40 negative correlations were found at the same significance level. Thirteen significant positive correlations were found between the bacterial genera and characteristics, while a significant negative correlation (*P* < 0.05) was found. No bacterial genus was correlated with TRP, PHE, total biogenic amines, trimethylpyrazine, and pH. Among the 13 significant positive correlations, *Lactobacillus* and Acetoin were the strongest (*r* = 0.959, *P* < 0.001), followed by *Acinetobacter* and 2,3-butanediol (*r* = 0.90, *P* < 0.01), *Aneurinibacillus* and SPM (*r* = 0.85, *P* < 0.01). The others included *Pseudomonas*, *Kurthia*, and PUT (rp = 0.67, rk = 0.71), with *Aneurinibacillus* and CAD (*r* = 0.74), *Lactobacillus* and HIS (*r* = 0.77), *Aneurinibacillus* and TYR (*r* = 0.79), *Citrobacter*, *Streptococcus*, *Kluyvera*, unclassified, and 2,3-butanediol (rc = 0.76, rs = 0.84, rk = 0.85, ru = 0.86), *Aneurinibacillus* and 3,5,6-ETMP (*r* = 0.79) showing weaker positive correlations (*P* < 0.05). *Aeromonas* was negatively correlated with 3,6-EDMP (*r* = –0.69, *P* < 0.05). As the highest abundance genus (Figure 3A), *Bacillus* was positively correlated with many chemical characteristics, such as SPD (*r* = 0.57, *P* = 0.09), SPM (*r* = 0.64, *P* = 0.06), 2,5-DMP (*r* = 0.57, *P* = 0.09), 3,6-EDMP (*r* = 0.58, *P* = 0.09), 3,5,6-ETMP (*r* = 0.61, *P* = 0.07), and tetramethylpyrazine (*r* = 0.58, *P* = 0.17), which indicated a positive correlation between *Bacillus* and the functionality of non-post fermented Shuidouchi.

As shown in Figure 5B, there were eight positive correlations between fungal genera and the chemical characteristics at a significance level of *P* < 0.1, while 33 negative correlations were found at the same significance. Four significant positive correlations were found between fungal genera and the assessed characteristics, while 14 significant negative correlations (*P* < 0.05) were found. No fungal genus was found to be correlated with PUT, total biogenic amines, 2,3-butanediol, 2,5-DMP, 3,6-EDMP, pH and temperature. Among the four significant positive correlations (*P* < 0.05), three positive correlations were found between *Tomentella* and TRP (*r* = 0.81), SPD (*r* = 0.71), trimethylpyrazine (*r* = 0.81), while one positive correlation was between unclassified and TYR (*r* = 0.72). Among the 14 significant negative correlations, unclassified *Pleosporales* and TYR showed the strongest correlation (*r* = –0.85, *P* < 0.01), while the others including *Anthopsis*, *Lactarius*, *Aspergillus*, *Mortierella*, *Guehomyces*, *Phallus*, and TYR (ra = –0.80, rl = –0.75, ra = –0.75, rm = –0.73, rg = –0.83, rp = –0.74), *Lactarius*, *Aspergillus*, *Mortierella*, *Guehomyces*, unclassified *Pleosporales* and SPM (rl = –0.69, ra = –0.80, rm = –0.70, rg = –0.75, ru = –0.71), *Aspergillus*, *Mortierella* and 3,5,6-ETMP (ra = –0.70, rm = –0.69) showed weaker positive correlations (*P* < 0.05). We found from the Pearson correlation heat map that many fungi were negatively correlated with TYR and SPM, indicating that the fungi could reduce the concentration of biogenic amines and improve the safety of the product.

### Analysis of Phylogenetic Investigation of Communities by Reconstruction of Unobserved States Functional Annotation

The KEGG functional annotation of the bacterial community is shown in Table 7. Seven primary functions were annotated during fermentation. The most abundant primary function was metabolism, which accounted for more than 30% of the reads in the samples. In addition, 39 KEGG pathways were annotated at level 2, including membrane transport, carbohydrate metabolism, amino acid metabolism, replication and repair, cellular processes, and signaling, poorly characterized, energy metabolism, metabolism of the cofactors and vitamins, translation, lipid metabolism, cell motility, metabolism, nucleotide metabolism, transcription, xenobiotic biodegradation, and metabolism, genetic information processing, enzyme families, signal transduction, folding, sorting and degradation, metabolism of terpenoids and polyketides, and the others (< 2%) were arranged in descending order of percentage. As shown in Table 7, the relative abundance of membrane transport, poorly characterized, metabolism, signal transduction, glycan biosynthesis and metabolism, biosynthesis of the other secondary metabolites, and infectious diseases decreased gradually. The relative abundance of amino acid metabolism, replication and repair, energy metabolism, metabolism of cofactors and vitamins, translation, lipid metabolism, cell motility, nucleotide metabolism, metabolism of terpenoids and polyketides, cell growth and death, endocrine system, transport and catabolism, neurodegenerative diseases, and environmental adaptation increased step by step. However, the relative abundances of other second level metabolic pathways were relatively stable.

**TABLE 7:** The KEGG functional annotation of bacterial 16S rDNA in the non-post fermented Shuidouchi.

KEGG pathways	Percentage composition in samples (%)						
	0 h	12 h	24 h	36 h	48 h	60 h	72 h
Cellular processes; cell growth and death	0.40	0.24	0.27	0.31	0.36	0.38	0.37
Cellular processes; cell motility	3.55	0.89	1.19	2.11	3.11	3.66	3.30
Cellular processes; transport and catabolism	0.27	0.16	0.17	0.21	0.25	0.27	0.26
Environmental information processing; membrane transport	11.61	18.92	18.00	15.88	13.43	12.87	13.38
Environmental information processing; signal transduction	2.36	2.45	2.39	2.30	2.15	2.05	2.05
Environmental information processing; signaling molecules and interaction	0.21	0.15	0.16	0.16	0.18	0.17	0.18
Genetic information processing; folding, sorting and degradation	2.14	1.96	1.99	2.02	2.03	2.00	1.99
Genetic information processing; replication and repair	7.26	5.46	5.80	6.27	6.96	7.13	7.04
Genetic information processing; transcription	3.10	3.31	3.24	3.22	3.17	3.21	3.21
Genetic information processing; translation	4.16	3.24	3.46	3.72	4.11	4.18	4.17
Human diseases; cancers	0.12	0.08	0.09	0.09	0.09	0.08	0.08
Human diseases; cardiovascular diseases	0.00^a^	0.00	0.00	0.00	0.00	0.00	0.00
Human diseases; immune system diseases	0.03	0.04	0.04	0.04	0.04	0.03	0.04
Human diseases; infectious diseases	0.28	0.51	0.50	0.42	0.35	0.32	0.34
Human diseases; metabolic diseases	0.06	0.06	0.06	0.06	0.07	0.07	0.07
Human diseases; neurodegenerative diseases	0.27	0.19	0.19	0.22	0.24	0.25	0.24
Metabolism; amino acid metabolism	10.56	8.79	8.91	9.47	10.13	10.36	10.19
Metabolism; biosynthesis of other secondary metabolites	0.61	0.79	0.77	0.70	0.64	0.60	0.63
Metabolism; carbohydrate metabolism	9.98	11.52	11.31	11.02	10.56	10.50	10.63
Metabolism; energy metabolism	4.91	4.63	4.65	4.85	5.04	5.24	5.16
Metabolism; enzyme families	2.52	2.02	2.03	2.07	2.11	2.01	2.00
Metabolism; glycan biosynthesis and metabolism	1.02	1.94	1.91	1.60	1.23	1.06	1.17
Metabolism; lipid metabolism	3.97	2.88	2.99	3.32	3.62	3.69	3.60
Metabolism; metabolism of cofactors and vitamins	3.90	3.82	3.82	4.01	4.20	4.43	4.36
Metabolism; metabolism of other amino acids	1.82	1.75	1.77	1.80	1.81	1.82	1.82
Metabolism; metabolism of terpenoids and polyketides	2.05	1.55	1.60	1.72	1.87	1.87	1.84
Metabolism; nucleotide metabolism	3.32	2.77	2.91	3.04	3.27	3.31	3.30
Metabolism; xenobiotics biodegradation and metabolism	3.28	2.84	2.86	2.90	2.94	2.79	2.81
Organismal systems; circulatory system	0.00^b^	0.00	0.00	0.00^c^	0.00	0.00	0.00
Organismal systems; digestive system	0.06	0.04	0.04	0.05	0.06	0.06	0.06
Organismal systems; endocrine system	0.30	0.16	0.18	0.23	0.29	0.32	0.30
Organismal systems; environmental adaptation	0.15	0.09	0.10	0.11	0.13	0.13	0.13
Organismal systems; excretory system	0.03	0.05	0.04	0.04	0.03	0.03	0.03
Organismal systems; immune system	0.06	0.04	0.04	0.04	0.03	0.02	0.02
Organismal systems; nervous system	0.06	0.05	0.05	0.06	0.06	0.06	0.06
Unclassified; cellular processes and signaling	5.90	5.33	5.24	5.42	5.52	5.57	5.46
Unclassified; genetic information processing	2.04	2.44	2.52	2.37	2.32	2.28	2.37
Unclassified; metabolism	2.47	3.46	3.34	2.94	2.55	2.31	2.45
Unclassified; poorly characterized	5.15	5.40	5.40	5.23	5.03	4.86	4.92

*^a^0.0004, ^b^0.0007, ^c^0.0002.*

**FIGURE 6:**
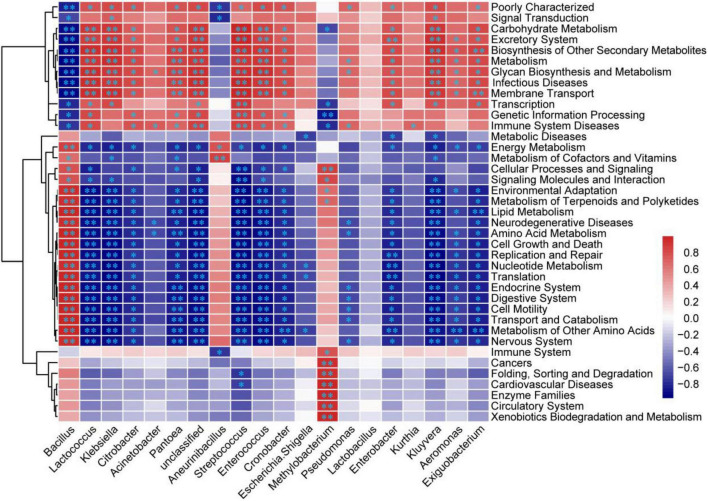
Heatmap of correlations between the bacteria and KEGG function pathways in the non-post fermented Shuidouchi. The Pearson correlation coefficient r between the bacteria and function pathway ranged from –1 to 1, where the positive correlation was *r* > 0, and the negative correlation was *r* < 0. *Represents *p* < 0.05 and ^**^represents *p* < 0.01.

The Pearson correlations between the relative abundances of the top 20 bacterial genera and the percentages of the 39 KEGG second level function pathways are shown in Figure 6. More than half of the correlations were significant, where the number of negative correlation coefficients was slightly more than the positive correlation coefficients. Among the top 20 bacteria, *Bacillus*, *Aneurinibacillus*, and *Methylobacterium* were positively correlated with amino acid metabolism, the metabolism of other amino acids, lipid metabolism, and the energy metabolism, while the other bacteria were positively correlated with carbohydrate metabolism, glycan biosynthesis and metabolism, biosynthesis of the other secondary metabolites, and metabolism. As the most dominant genus, *Bacillus* had complementary metabolic pathways with the other genera, as shown in Figure 6. The functional pathway analysis results indicated that the thermoduric and thermophilic bacteria such as *Bacillus* and *Aneurinibacillus* continued to reproduce at higher temperatures, which further caused the temperature of the product to increase to the limit value by the energy metabolism pathway. Meanwhile, the low abundance of bacterial genera decreased in the metaphase and anaphase stages of fermentation due to the high temperatures. They were prosperous in the initial stage of fermentation at moderate temperatures (Figure 3C), which was conducive to the formation of functional products by bringing about numerous metabolites into the product through the functional pathways of carbohydrate metabolism, glycan biosynthesis and metabolism, and the biosynthesis of other secondary metabolites (Figure 6).

## Discussion

Considering the bacterial communities in this research, we found that *Bacillus* was the most dominant bacterial genus in the fermentation process of the non-post fermented Shuidouchi, according to Illumina Miseq sequencing, and *Lactococcus* maintained a certain proportion in the different periods. *Klebsiella* was also found; however, it gradually decreased to low abundance in the metaphase and anaphase stages of fermentation. During the second half of fermentation (48–72 h), the diversity and relative abundance of the bacterial flora remained relatively steady, and the number of dominant bacterial genera decreased; however, the spore producing bacteria showed a diversified trend. In addition to *Bacillus*, *Aneurinibacillus* with a low relative abundance also appeared in the second half of fermentation. These results were similar to other studies on Shuidouchi. Chen et al. found that *Bacillus*, *Bacteroides*, and *Lactobacillus* were the main genera in Shuidouchi with post-fermentation from the different regions in China (9). Wang et al. found that the dominant bacteria were *Bacillus*, *Staphylococcus*, *Enterococcus*, *Proteus*, *Brevibacillus*, *Providencia*, *Weissella*, and *Ureibacillus* in wet and dry Enshi Douchi, and *Aneurinibacillus* also appeared in certain samples (36). The dominant bacteria in bacterial Douchi from Gansu province in China were *Bacillus* and *Ignatzschineria*, and *Aneurinibacillus* was also found in some samples (11). Further analysis of the bacterial community in Shuidouchi showed that the dominant bacteria were *Caldibacillus thermoamylovorans*, *Bacillus smithii*, *Bacillus licheniformis*, and *Bacillus subtilis*. Some harmful bacteria were also found, including *Proteus mirabilis*, *Serratia marcescens*, *Alcaligenes faecalis*, *Bacillus cereus*, which came from the condiments, such as spices, salt in Shuidouchi with post fermentation. The Shuidouchi with post-fermentation needs adding unsterilized condiments before post-fermentation at a room temperature. The harmful bacteria might enter the product with the unsterilized condiments, and the moderate temperature also accelerates the growth of these bacteria (10, 37, 38). The non-post fermented Shuidouchi in this research also contained *C. thermoamylovorans*, *B. licheniformis*, and *B. subtilis*, which was identified by the PCR-DGGE method and identification of the pure isolated cultures, while *Caldibacillus thermolactis* and *Ureibacillus thermosphaericus* were only detected by PCR-DGGE (data not shown); however, not by high-throughput sequencing in this research, which was possibly caused by different PCR methods (PCR-DGGE inspected the V6-V8 region of bacterial 16 S rDNA, while the bacterial high-throughput sequencing in this research inspected the V3-V4 region of 16 S rDNA). As a conclusion of this research and relative articles, the main bacterial genera in non-post fermented Shuidouchi were *Bacillus*, *Caldibacillus*, *Ureibacillus*, and *Aneurinibacillus*. The temperature had the greatest impact on the diversity of the bacterial flora in the non-post fermented Shuidouchi on the genus level (Figure 4C). At a moderate temperature in the initial stage of fermentation, the richness and evenness of the bacterial community increased; however, at a high temperature in the metaphase and anaphase stages of fermentation, only *Bacillus* grew normally, which reduced the richness and evenness of the bacterial communities in the samples (Figures 3A,C). *Bacillus* showed high levels of protease (39) and amino acid metabolism functionality (Figure 6) at high temperatures (45–50^°^C), which caused the pH to increase (Figure 5A and Table 1), reaching the growth limit of a variety of bacteria (Table 1). This further consolidated the absolute dominant position of the spore producing bacteria and promoted the diversification of spore producing bacteria.

The fungi in non-post fermented Shuidouchi during fermentation were much more diversified than the bacteria (Figure 2), and the richness and evenness of the fungal communities in the samples remained relatively constant compared to the bacterial communities (Figures 3B,D). We observed hundreds of fungal genera in the samples, such as unclassified, *Anthopsis*, unclassified fungi, *Lactarius*, unclassified *Agaricomycetes*, *Verticillium*, *Aspergillus*, *Fusarium*, unclassified *Atheliaceae*, *Tomentella*, and *Candida*. When the temperature was suitable, the fungi were prosperous and formed a mold type Douchi, which was the reason why so many types of Douchi in China were found to be mold type (24). However, in this non-post fermented Shuidouchi, the high temperatures suppressed fungal activity and prevented germination of the yeast cells, reproductive hyphae, spores and dormant hyphae, which caused a high diversity and evenness of the fungi in the different fermentation stages. As a result, the counts of culturable fungi were very low during fermentation (Table 2), among which the counts of yeast were lower than post fermented Shuidouchi due to non-post fermentation (9). The temperature was the environmental factor that had the greatest impact on the fungal communities of the non-post fermented Shuidouchi samples (Figure 4D), while high temperatures inhibited the growth of fungi. Consequently, the microbes in the non-post fermented Shuidouchi samples were mainly bacteria, and fungi only occupied a certain proportion when the temperatures at the beginning and end of fermentation were close to the appropriate temperature (Table 2). *Bacillus* potentially caused a pH increase in the product, which also inhibited the growth of fungi. Moreover, the volatiles generated by *Bacillus* could inhibit fungi such as *Botrytis cinerea* and *Phaeomoniella chlamydospora* by the function of alkylpyrazines (40, 41). *Bacillus* could also produce lipopeptide antibiotic iturin A, inhibiting fungi such as *Fusarium oxysporum* and *Rhizoctonia solani* (42). The former four conditions in the non-post fermented Shuidouchi formed a superimposed inhibitory effect on the fungi, especially on the molds.

Food born biogenic amines contain nitrogenous chemicals that originate from amino acids from microbial decarboxylation, and eight biogenic amines are often found. Some of these include polyamines such as PUT, SPM, and SPD, especially SPD, which have been found to exhibit protective effects against chronic diseases, such as metabolic disease, heart disease, neurodegeneration, and cancer (15, 16). PUT, SPM, and SPD were originally in cooked soybeans, and their quantities in non-post fermented Shuidouchi increased significantly to 11.42, 9.16, and 11.55 μg/g (Table 4), respectively. This would enhance the health protection functionality of the product, compared to cooked soybeans. The quantities of PUT, SPM, and SPD in this product were lower than Shuidouchi (12.21, 39.00, and 24.72 μg/g, respectively) and Natto (7.98, 45.48, and 339.70 μg/g, respectively). This led to a quantity of total biogenic amines that was far below the safety limit of 1,000 μg/g (16). The content of TRP in this product was 22.54 μg/g, which was nearly the same level as Shuidouchi and Natto, while the content of CAD was 22.54 μg/g, similar to Natto, but higher than Shuidouchi. PHE, HIS, and TYR were recognized as the toxic biogenic amines, monitored as safety indicators in foods. The content of PHE in this product was 8.44 μg/g, which was higher than Shuidouchi, but lower than the limit concentration of 30 μg/g. In this research, the maximum content of HIS and average content of TYR were 2.18 and 2.68 μg/g, respectively, lower than certain Natto and Shuidouchi products and far below the limit concentration (9, 16, 43, 44). *Bacillus* and *Aneurinibacillus* were positively correlated with a variety of bioamines (Figure 5A), mainly because these bacteria had strong amino acid metabolic functionality, secreting a large amount of protease (Figure 6). Some studies have found that *Bacillus subtilis* produced a large amount of toxic bioamines (15), while the content of biogenic amines did not exceed the safety limits in this study. This was most likely because many low abundance bacteria could efficiently use carbohydrate metabolism to produce various intermediate metabolites (Figure 6), which reacted with bioamines at high temperatures in the metaphase and anaphase stages of fermentation, reducing the concentration of bioamines *via* the Maillard reaction phenomena, such as Douchi browning. This improved the safety of the product. At the same time, polyamine quantity significantly increased during fermentation, resulting in greatly improved product functionality.

**FIGURE 7:**
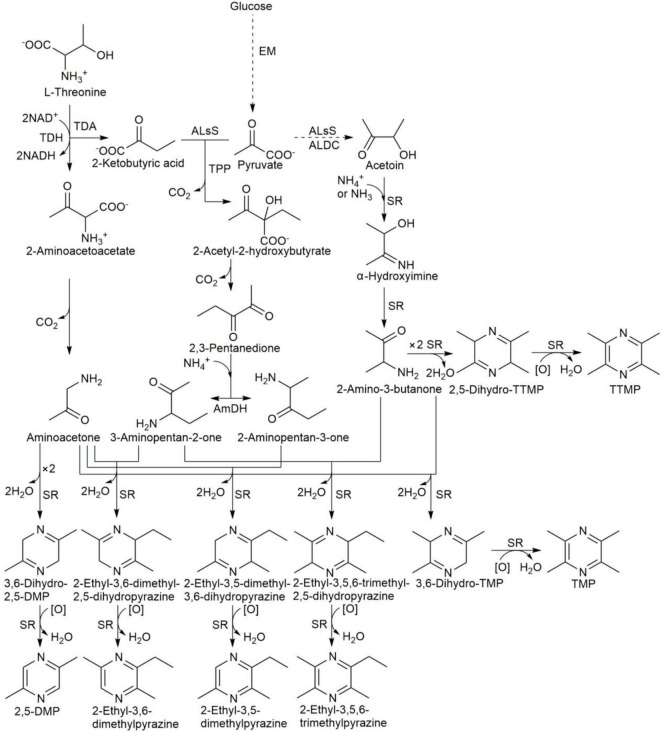
Synthesis pathways of alkylpyrazines in *B. subtilis*. EM, Embden-Meyerhof pathway; ALsS, acetolactate synthase; ALDC, α-acetolactate decarboxylase; TDH, L-threonine-3-dehydrogenase; TDA, threonine deaminase; AmDH, amine dehydrogenase; SR, spontaneous reaction under standard pressure and moderate or high temperature. The pathways were proposed by Zhang et al. (46, 47).

Alkylpyrazines are considered important flavor compounds and potential pharmacophores. Many bacteria can produce alkylpyrazines, such as *B. subtilis*, *B. licheniformis*, *Corynebacterium glutamicum*, *Paenibacillus polymyxa*, and *Serratia marcescens* (20–23, 45–47). *C. glutamicum* was also shown to produce a large amount of tetramethylpyrazine, trimethylpyrazine, 2, 3-dimethylpyrazine, 2-ethyl-3,6-dimethylpyrazine and a small amount of 2,5-dimethylpyrazine (45). In addition, *B. subtilis* was also shown to produce the above alkylpyrazines except for 2, 3-dimethylpyrazine (19). In our product, the same alkylpyrazines as *B. subtilis* were produced, in addition to 2, 3-dimethylpyrazine. *B. subtilis* was the dominant bacteria during the fermentation of non-post fermented Shuidouchi, and *Corynebacterium callunae* was found at a concentration of 7 log (CFU/g) only at 24 h during fermentation (data not shown). This indicated that *Corynebacterium* sp. could also benefit the production of 2, 3-dimethylpyrazine in this product, even as a small portion of bacteria. Tetramethylpyrazine, trimethylpyrazine, and 2, 5-DMP were the dominant alkylpyrazines, which correlated with the dominant bacteria such as *B. subtilis* and *B. licheniformis* in the non-post fermented Shuidouchi. The synthesis mechanisms of alkylpyrazines are proposed in Figure 7, according to the conclusions of Zhang et al. (46, 47). As the dominant bacteria, the species of *Bacillus* were positively correlated with amino acid metabolism and the metabolism of other amino acids (Figure 6), which included the dehydrogen reaction of amino acids such as L-threonine. This could be transformed to L-2-amino-acetoacetate by L-threonine-3-dehydrogenase, and further to aminoacetone by spontaneous reaction. Two molecules of aminoacetone could be combined to 3, 6-dihydro-2,5-DMP, transforming into 2,5-DMP spontaneously. Many bacteria were significantly correlated with carbohydrate metabolism, as well as glycan biosynthesis and the metabolism pathway, which caused the hydrolysis of polysaccharides and the formation of acetoin by the EM pathway (Figures 6, 7). Thus, acetoin reacted with ammonia or ammonium (originating from amino acid metabolism) to form α-hydroxyimine, and further 2-amino-3-butanone spontaneously. Two molecules of 2-amino-3-butanone could condense to 2,5-dihydro-TTMP, transforming to tetramethylpyrazine spontaneously. This chemical reaction would then be accelerated by higher temperatures (20); thus, tetramethylpyrazine accumulated quickly during high temperature fermentation (Table 6), improving the health protection functionality of the product. The initial stage of fermentation was also important because of its moderate temperature, which was suitable for the growth of most types of bacteria and the accumulation of fermentable sugar, accelerating the formation of acetoin by the carbohydrate metabolism pathway (Figure 6). Acetoin could enter the environment outside the bacterial cells, reacting with ammonia or ammonium to form 2-amino-3-butanone, and 2-amino-3-butanone and aminoacetone could form 3,6-dihydro-TMP, transforming into trimethylpyrazine spontaneously (Figure 7). Tetramethylpyrazine, 2,5-DMP, and trimethylpyrazine were the alkylpyrazines with the highest content in the non-post fermented Shuidouchi, while 2-ethyl-3,6-dimethylpyrazine and 2-ethyl-3,5,6-trimethylpyrazine had less content (Table 6). This was attributed to the excessive amount of ammonium ions that were produced by *Bacillus* through amino acid metabolism, caused the pH to increase (Table 1), and bacterial cells had to convert pyruvate into acetoin to more effectively consume the ammonium, while the efficiency of the 2,3-pentanedione pathway was too low because one ammonium ion consumed two pyruvate molecules (Figure 7). The successive inoculation of *Pichia kudriavzevii*, *Rhizopus* sp., and *B. licheniformis* increased the production of tetramethylpyrazine in the Chinese liquor. Thus, lowering the temperature of initial fermentation could be adopted as a method to facilitate the growth of more fungi, potentially increasing tetramethylpyrazine production due to the high hydrolase activity of the fungi for the polysaccharides (48).

The processing of non-post fermented Shuidouchi is a heat preservation process (Section “Non-post Fermented Shuidouchi Fermentation and Sampling”). It first involves a two-stage temperature control strategy [moderate temperature (35–42^°^C) before a high-temperature (52^°^C) stage], which could be observed from spontaneous fermentation. Before a timepoint of 24 h of fermentation (initial stage of fermentation), the temperature increased slowly (Table 1), and the bacterial community structures were consequently similar between 12 and 24 h (Figure 4A). The genera of *Bacillus*, *Lactococcus*, *Klebsiella*, *Citrobacter*, *Acinetobacter*, and *Pantoea* were more than 1% of the relative abundance in the initial stage with moderate temperature (Table 1 and Figure 3A). However, the temperature remained high (45–52^°^C) after a timepoint of 24 h (Table 1). The high temperature caused the relative abundance of *Bacillus* and *Aneurinibacillus* to increase, accompany with increasing pH value (Table 1 and Figure 3A). The genera of *Bacillus*, *Lactococcus*, *Klebsiella*, and *Aneurinibacillus* remained more than 1% of the relative abundance after a timepoint of 24 h of fermentation (Figure 3A). These changes in the bacterial community structure caused the changes in bacterial metabolic pathways at the two main temperature stages. Before a timepoint of 24 h during fermentation, the percentage composition of carbohydrate metabolism was higher than at the stage after a timepoint of 24 h (Table 7). However, the percentage compositions of amino acid metabolism, other amino acids metabolism, and lipid metabolism were higher after a timepoint of 24 h during fermentation (Table 7). Combined with high temperature, these changes in metabolisms caused the accumulations of the functional metabolites such as alkylpyrazines and polyamines, as discussed previously. Metagenomic (49) and metatranscriptomic (50) analyses should be adopted in further research, for a more comprehensive and in-depth understanding of the fermentation process. Some researchers had used functional microbe to increase the antioxidant activity of fermented soybeans by solid-state fermentation (51). We have isolated the cultured microbes in non-post fermented Shuidouchi, and the functional microbes will be screened to increase the multifunctionality of the product.

In conclusion, non-post fermented Shuidouchi was confirmed as bacterial type Douchi due to the high counts of bacteria and very low counts of fungi, as determined during fermentation. The bacterial genus *Bacillus* was the most dominant microbe throughout the fermentation process, and high counts of species such as *B. subtillus* could improve the probiotic functionality of this product. The temperature was the most important influential factor for the microbial diversity of the samples, and the succession of microbial communities brought about changes in the characteristic metabolites. Furthermore, a two-stage temperature control strategy [moderate temperature (35–42^°^C) at first, then at a high temperature (52^°^C)] could be summarized from this spontaneous fermentation. This strategy could control the concentration of biogenic amines within the safety limit, and significantly increase the concentration of polyamines such as SPD, which offer multiple protective health effects. This strategy could also increase the types and concentrations of alkylpyrazines, especially tetramethylpyrazine, which has shown a functional effect on human cardiovascular and cerebrovascular health. This two-stage temperature control strategy could be applied to the industrial production of non-post fermented Shuidouchi to accelerate the function of fermented soybeans. Meanwhile, this strategy was low cost and could be widely popularized in households everywhere, offering a worldwide solution to human hunger and nutrition issues.

## Data Availability Statement

The datasets presented in this study can be found in online repositories. The names of the repository/repositories and accession number(s) can be found below: BioProject: PRJNA830651; PRJNA830678; https://www.ncbi.nlm.nih.gov/.

## Author Contributions

YC and FQ designed and conducted this research, performed the experiments, and completed the manuscript. MD acquired funding, designed the experimental scheme, supervised the experiments, and revised the manuscript. All authors contributed to the article and approved the submitted version.

## Conflict of Interest

The authors declare that the research was conducted in the absence of any commercial or financial relationships that could be construed as a potential conflict of interest.

## Publisher’s Note

All claims expressed in this article are solely those of the authors and do not necessarily represent those of their affiliated organizations, or those of the publisher, the editors and the reviewers. Any product that may be evaluated in this article, or claim that may be made by its manufacturer, is not guaranteed or endorsed by the publisher.

## Footnotes

1
http://drive5.com/usearch

2
http://drive5.com/usearch/manual/uchime_algo.html

3
http://rdp.cme.msu.edu

4
http://rdp.cme.msu.edu/misc/resources.jsp

5
http://www.arb-silva.de

6
http://unite.ut.ee/index.php

7
https://mothur.org
